# Analysis of Measles-Mumps-Rubella (MMR) Titers of Recovered COVID-19 Patients

**DOI:** 10.1128/mBio.02628-20

**Published:** 2020-11-20

**Authors:** Jeffrey E. Gold, William H. Baumgartl, Ramazan A. Okyay, Warren E. Licht, Paul L. Fidel, Mairi C. Noverr, Larry P. Tilley, David J. Hurley, Balázs Rada, John W. Ashford

**Affiliations:** a World Organization, Watkinsville, Georgia, USA; b Nevada Spine Center, Las Vegas, Nevada, USA; c Kahramanmaraş Sütçü İmam University, Kahramanmaraş, Turkey; d Warren Alpert Medical School of Brown University, Providence, Rhode Island, USA; e Louisiana State University Health Sciences Center, New Orleans, Louisiana, USA; f Tulane University School of Medicine, New Orleans, Louisiana, USA; g VetMed Consultants, Inc., Santa Fe, New Mexico, USA; h College of Veterinary Medicine, University of Georgia, Athens, Georgia, USA; i Stanford University, Stanford, California, USA; Albert Einstein College of Medicine

**Keywords:** coronavirus, COVID-19, immunization, measles, MMR, mumps, rubella, SARS-CoV-2, titers, vaccines

## Abstract

The measles-mumps-rubella (MMR) vaccine has been theorized to provide protection against coronavirus disease 2019 (COVID-19). Our aim was to determine whether any MMR IgG titers are inversely correlated with severity in recovered COVID-19 patients previously vaccinated with MMR II. We divided 80 subjects into two groups, comparing MMR titers to recent COVID-19 severity levels. The MMR II group consisted of 50 subjects who would primarily have MMR antibodies from the MMR II vaccine, and a comparison group of 30 subjects consisted of those who would primarily have MMR antibodies from sources other than MMR II, including prior measles, mumps, and/or rubella illnesses. There was a significant inverse correlation (*r_s_* = −0.71, *P* < 0.001) between mumps virus titers (mumps titers) and COVID-19 severity within the MMR II group. There were no significant correlations between mumps titers and severity in the comparison group, between mumps titers and age in the MMR II group, or between severity and measles or rubella titers in either group. Within the MMR II group, mumps titers of 134 to 300 arbitrary units (AU)/ml (*n* = 8) were found only in those who were functionally immune or asymptomatic; all with mild symptoms had mumps titers below 134 AU/ml (*n* = 17); all with moderate symptoms had mumps titers below 75 AU/ml (*n* = 11); all who had been hospitalized and had required oxygen had mumps titers below 32 AU/ml (*n* = 5). Our results demonstrate that there is a significant inverse correlation between mumps titers from MMR II and COVID-19 severity.

## Abstract (precis)

COVID-19 has presented various paradoxes that, if understood better, may provide clues to controlling the pandemic, even before a COVID-19 vaccine is widely available. First, young children are largely spared from severe disease. Second, numerous countries have COVID-19 death rates that are as low as 1% of the death rates of other countries. Third, many people, despite prolonged close contact with someone who is COVID-19 positive, never test positive themselves. Fourth, nearly half of people who test positive for COVID-19 are asymptomatic. Some researchers have theorized that the measles-mumps-rubella (MMR) vaccine may be responsible for these disparities. The significance of our study is that it showed that mumps titers related to the MMR II vaccine are significantly and inversely correlated with the severity of COVID-19-related symptoms, supporting the theorized association between the MMR vaccine and COVID-19 severity.

## INTRODUCTION

It has been theorized that the measles-mumps-rubella (MMR) vaccine may protect against or reduce the severity of coronavirus disease 2019 (COVID-19) infection (1–5). Gold et al. introduced this theory in March 2020 after observing that recent, large-scale MMR vaccination campaigns were associated with countries with the fewest COVID-19 deaths (6). We set out to investigate whether measles, mumps, or rubella IgG titer tests would reveal an inverse correlation between antibody concentrations from MMR II vaccinations and COVID-19 severity.

Vaccines induce a variety of different antibodies to protect against a virus, since each part of an antigen stimulates different antibodies. Titer tests determine seropositivity based upon a narrow set of antibody concentrations but do not measure the viral neutralization power of all the different antibodies related to a virus. Since only a narrow subset of antibodies related to measles, mumps, or rubella may protect against COVID-19, one must consider the possibility that even if MMR II is cross-protective against COVID-19, titer tests alone may not paint a complete picture.

We had to consider the various ways in which individuals develop MMR-related antibodies, as each would affect titer values differently. First, individuals may have antibodies from the current MMR II vaccine by Merck, initially licensed in 1979, which includes the Edmonston strain of measles, the Jeryl Lynn (B-level) strain of mumps, and the Wistar RA 27/3 strain of rubella (7). Second, individuals may have antibodies from early monovalent measles, mumps, or rubella vaccines. Third, individuals may have antibodies from other combination vaccines, including the original MMR vaccine by Merck, utilizing the less effective HPV-77 DE-5 strain of rubella (8). Lastly, older adults, including virtually all born before 1957, most likely have MMR antibodies from naturally acquired infections (9). We also had to consider that if someone was at some point infected by the measles virus itself, that individual may have had up to 73% of their prior antibody repertoire eliminated (10).

## RESULTS

### Mumps titers were inversely correlated with severity and symptom scores.

**FIG 1:**
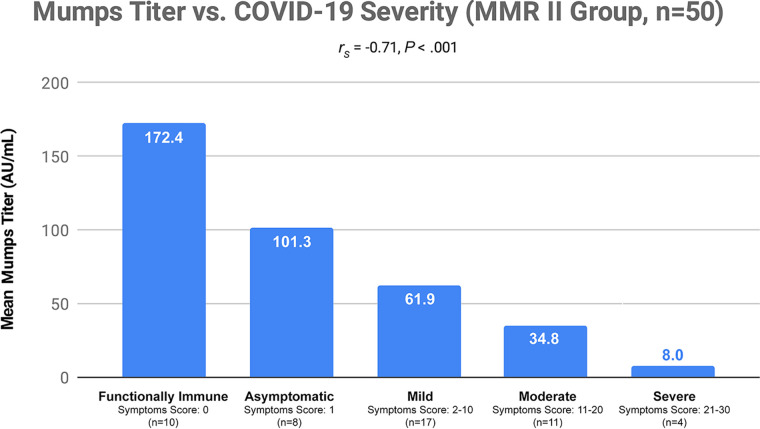
Mean mumps titer values (in arbitrary units per milliliter) were compared to each of five severity categories. Each severity category was based upon the symptom scores shown in Fig. 2. “Functionally Immune” data represent subjects with a severity score of 0. “Asymptomatic” data represent those with a score of 1, i.e., those who were COVID-19 positive but had no symptoms. “Mild” data represent those with scores ranging from 2 to 10. “Moderate” data represent those with scores ranging from 11 to 20. “Severe” data represent those with scores from 21 to 30. A *P* value of less than 0.05 was considered to indicate statistical significance, with an adjustment made for three comparisons. Severity data were not normally distributed, so comparisons were done with a Spearman’s rank correlation coefficient.

**FIG 2:**
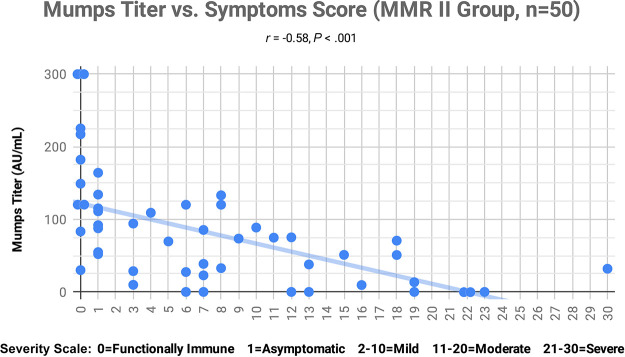
Mumps titer values (AU/ml) for each subject were plotted against symptom scores, with overlapping scores subjected to dithering. Each subject began with a score of zero, and then points were added. One point was added for each of the following symptoms: COVID-19 positivity, dry cough, sore throat, slight shortness of breath, headache, confusion, muscle aches/pain, fever over 101° F, nausea and/or vomiting, or diarrhea. Two points were added for each of the following symptoms: severe difficulty breathing, chest pain, or sudden loss of sense of smell/taste. Five points were added for each of the following statuses: hospitalization, requirement of supplemental oxygen, or intubation on a ventilator. A *P* value of less than 0.05 was considered to indicate statistical significance, with an adjustment made for three comparisons. All data were normally distributed, so comparisons were done with a Pearson’s correlation coefficient.

We found a significant inverse correlation (*r_s_* = −0.71, *P* < 0.001) between COVID-19 severity and mumps IgG titers (Fig. 1). There was also a significant inverse correlation (*r_s_* = −0.58, *P* < 0.001) between symptom scores used to determine COVID-19 severity and mumps IgG titers (Fig. 2). There were no significant correlations between mumps virus titers (mumps titers) and severity, or between mumps titers and symptom scores, in the comparison group. There were also no significant correlations between severity or symptom scores, and measles or rubella titers, in either group (Table 1).

**TABLE 1:** MMR IgG titers versus severity and symptom scores^*a*^

Group	Severity	Symptom score
MMR II (*n* = 50)		
Measles	*r_s_* = −0.08, *P *= 0.93	*r* = −0.14, *P *= 0.69
Mumps	***r_s_* = −0.71, *P *<** **0.001**	***r* = −0.58, *P *<** **0.001**
Rubella	*r_s_* = −0.21, *P *= 0.37	*r* = −0.18, *P *= 0.51

Comparison (no MMR II, *n* = 30)		
Measles	*r_s_* = 0.19, *P *= 0.70	*r *= 0.07, *P *= 0.98
Mumps	*r_s_* = 0.22, *P *= 0.56	*r *= 0.14, *P *= 0.86
Rubella	*r_s_* = 0.16, *P *= 0.78	*r *= 0.17, *P *= 0.75

a *P* values were adjusted for 3 comparisons. Values with bold highlighting indicate statistically significant results.

Mumps titer values in the immunoassay (IA) that we used could range from a minimum of 0 (seronegative) to 300 (maximum seropositivity) arbitrary units (AU)/ml. The following observations were noted in the MMR II group: mumps titers of 134 to 300 AU/ml were found only in those who had asymptomatic COVID-19 cases or who were functionally immune (*n* = 8); all who had mild COVID-19 cases had mumps titers below 134 AU/ml (*n* = 17); all who had moderate COVID-19 symptoms had mumps titers below 75 AU/ml (*n* = 11); all who had been hospitalized and had required oxygen therapy had mumps titers below 32 AU/ml (*n* = 5).

In the MMR II group, 5 of 50 subjects had mumps titers of 182 AU/ml or above, and all 5 of these subjects, ranging in age from 21 to 41, were functionally immune. Functionally immune subjects tested negative in severe acute respiratory syndrome coronavirus 2 (SARS-CoV-2) nasopharyngeal and/or SARS-CoV-2 antibody tests. Each had had several days of extensive exposure to an actively symptomatic person who was positive for SARS-CoV-2, e.g., a housemate or a spouse. These subjects took no social distancing or other precautions such as wearing masks. Despite this, the functionally immune subjects never tested positive for COVID-19 despite the ease of transmission of SARS-CoV-2.

### The inverse correlation between mumps titers and severity was not age related.

No significant correlations were observed between age and mumps, measles, or rubella titer values in the MMR II group (Table 2). There were no significant correlations between age and severity levels (*r_s_* = −0.06, *P = *0.69), or between age and symptom scores (*r *= 0.04, *P = *0.77), in the MMR II group. There were no significant correlations between age and severity (*r_s_* = −0.14, *P = *0.47), or between age and symptom scores (*r* = −0.13, *P = *0.48), in the comparison group.

**TABLE 2:** MMR IgG titers versus age^*a*^

Group	Age
MMR II (*n* = 50)	
Measles	*r* = −0.28, *P *= 0.13
Mumps	*r *= 0.13, *P *= 0.77
Rubella	*r *= 0.17, *P *= 0.56

Comparison (no MMR II, *n* = 30)	
Measles	***r *=** **0.48, *P *=** **0.02**
Mumps	***r *=** **0.49, *P *=** **0.02**
Rubella	***r *=** **0.44, *P *=** **0.04**

a *P* values were adjusted for 3 comparisons. Values with bold highlighting indicate statistically significant results.

### COVID-19 case prevalence is seven times lower in young children than in adults.

**FIG 3:**
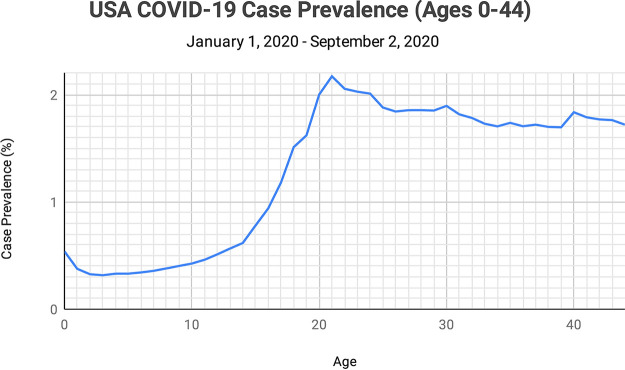
COVID-19 case totals provided by the CDC, at individual years of age 0 through 44, for the United States (1 January 2020 through 2 September 2020), were each divided by the total estimated 2020 U.S. population for each individual age to determine prevalence percentages.

Publicly available case prevalence data are typically published only in large increments of years of age, e.g., “<20,” “21 to 29,” and “30 to 39.” Through an open records act request, the Centers for Disease Control and Prevention (CDC) provided us with COVID-19 case totals by individual years of age. We divided the total number of cases for each individual age by the total estimated 2020 population of the United States for each individual age to determine the prevalence percentages by age for cases (Fig. 3). Prevalences of positive cases began to rise slowly at age 5, began to rise most sharply at age 14, and then peaked at age 21 at 2.17% prevalence, a rate seven times higher than that determined for the youngest ages.

After two MMR II vaccinations 5 years apart, IgG titers for rubella remained seropositive in 93% of individuals, IgG titers for measles remained seropositive in 82% of individuals, and IgG titers for mumps remained seropositive in 40% of individuals (11). As such, the mumps titer is the only MMR titer which steadily and substantially decreases over time after vaccination, decreasing 9.2% per year (12). On the basis of a 9.2% mean annual decay rate for mumps titers, and 300 AU/ml being the maximum seropositive value for mumps titers in our study, we calculated that an individual’s mean mumps titer would decrease to 142 AU/ml 9 years after vaccination with MMR II and to 130 AU/ml after 10 years. On the basis of the fact that the mean age for the second vaccination of MMR II for children in the United States is 5 years, the mean age at which a child’s mumps titers would decrease below 134 AU/ml would be 14 years.

## DISCUSSION

We found that high mumps titers (134 to 300 AU/ml) from MMR II vaccinations were found only in subjects with asymptomatic and functionally immune COVID-19 cases. Subjects with moderate and severe cases of COVID-19 all had low mumps titer values (below 75 AU/ml). The significant inverse correlations that we observed in the MMR II group between mumps titers and severity, as well as between mumps titers and symptom scores, indicate that there is an association between mumps titers and COVID-19. This significant inverse correlation existed at all ages. In contrast, similar associations were not identified for measles or rubella titers. Prior research had suggested a possible relationship between COVID-19 and measles or rubella, in addition to mumps, based on the sequence homology of each with SARS-CoV-2 (13). Our findings, however, have placed more emphasis on mumps.

There were no significant correlations in the MMR II group between age and severity or symptom scores, nor were there any significant correlations between age and titer values. COVID-19 severity levels were represented across all ages, eliminating the possibility that the inverse correlations that we observed between mumps titers and severity were confounded by the ages of study subjects or age-associated factors such as the prevalence of comorbidities. For example, three subjects in the MMR II group with severe cases were 28 to 33 years of age and showed low mumps titers ranging from 0 to 31.9 AU/ml, while three other subjects in the MMR II group who were 38 to 41 years of age were functionally immune, with mumps titers ranging from 120 to 300 AU/ml. These observations indicate either that some older subjects in the MMR II group retained high antibody concentrations from MMR II vaccinations given to them as children or that some may have received MMR II boosters as adults. MMR II boosters are often given to persons entering the military, or to women of child-bearing age. A limitation of our study was that the nonrandom process of selection of applicants employed to ensure a wide variety of severity levels over a wide age range had the potential to introduce biases.

Although our study showed no correlation between rubella titers or measles titers and COVID-19 severity, if some portion of measles or rubella antibodies protects against COVID-19, measles or rubella titer seropositivity tests may not measure those antibody isoforms. Hence, while our study provided clear evidence linking mumps seropositivity to COVID-19 severity, we do not dismiss the possibility that there are links between measles or rubella seropositivity that have not yet been identified.

Taken together, our finding that 14 years is the mean age at which mumps titers fall below 134 AU/ml and our finding that mumps titers above this value (in the MMR II group) were exclusively associated with functionally immune and asymptomatic individuals suggest that age 14 years would be the pivot point after which a further decline in mumps titers would be associated with a sharp rise in age-related risk of a COVID-19-positive test result or having a symptomatic case. The CDC data that we reviewed indeed indicated that the age of 14 years is the pivot point at which both the incidence of COVID-19-positive cases and the risk of death begin to rise sharply. We acknowledge that the sharp rise in the incidence of cases at age 14 years may also be influenced by other factors; however, this association adds further support to the hypothesis that MMR II-induced immunity may be a significant factor in protecting vaccinated children through age 14 years from COVID-19, in addition to protecting older adults with adequate mumps titers.

In the United States, there have been 65% more COVID-19 cases diagnosed in infants less than 12 months of age than in children 2 years of age. The increase in the number of cases in infants less than 12 months of age might also be related to an association between MMR II and COVID-19 because infants do not receive their first MMR II vaccination until 12 to 15 months of age. Those infants who are protected might be protected by transplacentally acquired MMR antibodies lasting up to 6 months of age (14). A study in China further supported age-related associations in children. While COVID-19-positive children aged 1 to 15 years were found to be asymptomatic 3.1% to 6.5% of the time, infants less than 1 year of age in China were asymptomatic only 1.9% of the time. Further, children in that Chinese study who were aged 1 to 15 years had critical COVID-19 illness no more than 0.7% of the time, while those less than 1 year of age had critical illness 1.9% of the time (15).

The lack of correlation between mumps titers and severity or symptom scores in the comparison group does not mean that MMR II is the only source of antibodies that may confer protection against COVID-19. It is possible that the original MMR vaccine, other combination vaccines, prior monovalent vaccines, and prior infections with measles, mumps, and/or rubella may also confer some level of protection from COVID-19. Such associations, however, may be impossible to detect through titer tests alone since older people with naturally acquired mumps, measles, or rubella antibodies usually have high titer values for the measured antibodies, which may not be the same ones relevant to protection from COVID-19. Such high titers most often are indicative of naturally acquired antibodies, not those from vaccinations (16). All titers significantly and positively correlated with age in the comparison group, indicating that older subjects were more likely to have antibodies from natural infections and not from vaccinations.

Since the presence of high mumps titers did not indicate a level of protection from COVID-19 in those who have not had the MMR II vaccine, if MMR II is given in a trial to evaluate possible protection against COVID-19, it should be given regardless of mumps titer or other MMR titer seropositivity, particularly in older adults. Given the significance of our findings related to mumps titers, it is also important to emphasize that while most MMR vaccines worldwide use the same strains of measles and rubella as MMR II from Merck, at least 10 different mumps strains have been used in recent decades by other manufacturers of MMR (17). In addition to Jeryl Lynn, the most common mumps strains currently in use in vaccines manufactured outside the United States are RIT 4385, Urabe, and l-Zagreb.

Despite many older adults having high MMR seropositivity from naturally acquired antibodies, the underlying antibodies that may protect against COVID-19 may have waned beyond protective levels. Further, there are several different wild-type mumps strains in circulation (18) and, should the Jeryl Lynn mumps strain in the MMR II vaccine be found to be protective against COVID-19, this does not mean all mumps strains would be protective. Natural infections that yielded long-lasting, high titers would often have been quite severe and broadly systemic, causing several rounds of affinity maturation. This level of affinity maturation, leading to a much narrower population of strongly recognized antigens, is different from the brief and limited replication that vaccines provide in generating adaptive immunity. Advanced test methods, such as VirScan, could possibly provide more definitive information (19).

While the associations that we have observed between MMR II and COVID-19 do not prove causation, the significant associations lend further support to the theory that the MMR II vaccine may provide long-term, cross-protective immunity against COVID-19. A possible factor in this protection is the sequence homology between both mumps and measles viruses and the fusion proteins of SARS-CoV-2 and/or the 29% amino acid sequence homology between the rubella virus and Macro (ADP-ribose-1-phosphatase) domains of SARS-CoV-2 (13). This may provide a memory target for adaptive immunity that leads to rapid, but relatively weak, proinflammatory or suppressor/regulator cytokine upregulation. This may then modulate early innate immune activity and invariant T cell activity and begin priming memory B cells for antibody production. It is likely that immunity induced by severe clinical infection with rubella would provide a long-lived memory pool of T cells that could be reactivated years after the infection. Vaccine-induced memory cells also appear to have a relatively long duration in the body, often 7 years or more, with smallpox vaccine memory documented to last at least 88 years (20).

There are other ways in which the MMR II vaccine may function against COVID-19. Live attenuated vaccines induce forms of nonspecific trained innate immunity that may act against COVID-19. The term “trained innate immunity” is based upon observations made in different infection and vaccination models describing increased resistance to reinfection independent of memory lymphocyte reactivation, resulting in the hypothesis that the innate immune system “remembers” prior infections through cellular epigenetic reprogramming (21). Studies of trained innate immunity related to Mycobacterium bovis BCG have found that a heterologous T cell immunological phenotype can last from 3 months to 1 year and that the heterologous protection against infection can last up to 5 years (22, 23). Furthermore, recent reports have proposed transgenerational effects related to innate immune memory (24, 25). Even keeping this in mind, trained innate immunity is generally considered reversible and shorter-lived than antigen-specific, adaptive immune memory (26).

T cell reactivation occurs when weak to moderate affinity binding occurs in the proper major histocompatibility complex (MHC) context and is enhanced when local activation of NK cells (or other interferon-producing cells) in the infection target tissue are triggered (27). We believe that the epiphenomenal model, involving differentially assorted MHC genes governing the response, is not as likely as the biologically driven model of low-level cross-reactivation of memory cells combined with trained innate immunity. This is due to the distribution of the individuals in the population who are resistant. On a gene distribution basis, assortment of MHC class I and II alleles in the U.S. population does not follow the population split observed here (28, 29).

An appropriate, trained innate immune response due to exposure to prior live vaccines such as MMR II improving the symptoms of COVID-19 also provides an attractive, potential explanation because the success of SARS-CoV-2 is largely attributed to its ability to evade the early, antiviral innate immune clearance mechanisms and to exaggerate innate immune responses in late stages of the infection leading to the cytokine storm, a key component of COVID-19-related acute respiratory distress syndrome (30, 31). SARS-CoV2 is known to suppress interferon production, evade natural killer cell-mediated cytotoxicity, and overstimulate the NLRP3 inflammasome, all of which are essential antiviral innate immune mechanisms that have also been implicated in the mechanism of trained innate immunity (26). Exposure to strong bacterial or viral vaccine antigens also appears to induce metabolic reprogramming events modifying enzyme activity and histone packing to improve the response to other challenges using similar inflammatory and immune activation signaling networks (32). Accordingly, clinical trials are being conducted to determine whether MMR II can induce immune-tolerant myeloid-derived suppressor cells that inhibit sepsis, the most severe life-threatening symptom of COVID-19 infection (33).

If MMR II is proven to be effective against COVID-19 in the short or long term, preclinical and postclinical management of COVID-19 infections will certainly be impacted. The association between MMR II and COVID-19 may also warrant consideration during development and testing of monovalent COVID-19 vaccines, as a patient’s previous immune status, including prior vaccinations, may need to be considered when evaluating disease prevention (34). Suggestions for further research include randomized controlled clinical trials of MMR II, investigations of anti-mumps antibodies to assess potential effects against SARS-CoV-2, the utilization of a larger sample size, and employing more-predictive types of analysis of data to establish a causal link between various levels of immunity offered by MMR II and severity of COVID-19 symptoms.

## MATERIALS AND METHODS

### Study design.

MMR IgG titers were measured in 80 adults who had consented to join our study. All were born in the United States and were over 18 years of age. Applicants (*n* = 568) were screened using a Health Insurance Portability and Accountability Act (HIPAA)-compliant online form in which informed consent was obtained, approved by our Investigational Review Board (Integrity IRB Protocol identifier [ID]: 40005). Subjects were chosen from applicants who responded to online advertisements that we ran seeking recovered COVID-19 patients for this study. Each applicant was required to upload documentation of their COVID-19 medical history, including COVID-19 test results and hospitalization records, as well as to complete an in-depth online survey in which they provided details related to their COVID-19 symptoms and outcomes on a HIPAA-compliant form. Follow-up was done as necessary to verify the information provided to ensure that each subject met the study criteria. Subjects in the study were selected in the order in which they had applied, if they matched criteria for either the MMR II group or the comparison group, until all 80 subjects had been selected and tested. Review of applicants stopped once 80 subjects had been selected. The selection and titer test process ran from 12 May 2020 through 26 August 2020. A small stipend to help defray costs was available to subjects. We divided the subjects into two groups.

The first group was the MMR II group, which consisted of 50 subjects (33 women and 17 men; mean age, 30.6 years [standard deviation {SD}, 7.6 years]) whose only likely source of MMR antibodies would have been the MMR II vaccine. Of these, 40 had previously had COVID-19-positive test results, with statuses ranging from asymptomatic to requiring a ventilator, and 10 subjects were functionally immune (COVID-19 negative despite strong COVID-19 exposure). Functionally immune subjects had had several days of close contact with someone who was symptomatic and who had tested positive for COVID-19, without either person social distancing or wearing masks. Despite the extensive contact, the functionally immune subjects tested negative for COVID-19 and never exhibited symptoms. Each person in the MMR II group was U.S. born and either had been born on or after 1 December 1978, meaning that that individual would have received the first MMR vaccination after the MMR II vaccine was launched, or, if born on or after 1 December 1973 but before 1 December 1978, had been specifically documented to have received one or more MMR II vaccinations.

The remaining 30 subjects (18 women and 12 men; mean age, 57.4 years [SD, 7.8 years]) made up the members of the comparison group, all of whom tested positive for COVID-19 and had been born before 1 December 1976. All subjects in the comparison group had birth dates at least several years before the MMR II vaccine was launched, and none had any record of ever having received an MMR II vaccination or booster. The age disparity between the MMR II group and the comparison group was purposeful, which is why we utilized a comparison group, not a control group. Age differentiation was the only way to accurately separate people who definitively had prior MMR II vaccinations from those who had not.

Mumps, measles, and rubella IgG titers were measured by Quest Diagnostics using Liaison analyzers with chemiluminescence immunoassay (CLIA) technology for the qualitative determination of IgG antibodies in human serum specimens. The method for qualitative determination of each specific IgG corresponding to each virus was an indirect CLIA. The principal components of each test were magnetic particles (solid phase) coated with recombinant antigen and a conjugate of mouse monoclonal antibody to human IgG linked to an isoluminol derivative (isoluminol-antibody conjugate). Diagnostic sensitivities were as follows: measles, 94.7% (95% confidence interval [CI], 91.7% to 96.9%); mumps, 98.5% (95% CI, 96.5% to 99.5%); rubella, 100% (95% CI, 99.3% to 100%). Diagnostic specificities were as follows: measles, 97.4% (95% CI, 94.1% to 99.2%); mumps, 98.2% (95% CI, 94.8% to 99.6%); rubella, 100% (95% CI, 97.0% to 100%).

### Symptom score calculations.

Each subject began with a score of zero, and then points were added. One point was added for each of the following symptoms: COVID-19 positivity, dry cough, sore throat, slight shortness of breath, headache, confusion, muscle aches/pain, fever over 101° F, nausea and/or vomiting, or diarrhea. Two points were added for each of the following symptoms: severe difficulty breathing, chest pain, or sudden loss of sense of smell/taste. Five points were added for each of the following statuses: hospitalization, requirement of supplemental oxygen, or intubation on a ventilator.

### Severity levels.

Five severity levels were designated based upon symptom scores. “Functionally Immune” data represent subjects with a symptom score of 0. “Asymptomatic” data represent those with a symptom score of 1, i.e., those who were COVID-19 positive but had no symptoms. “Mild” data represent those with symptom scores ranging from 2 to 10. “Moderate” data represent those with symptom scores ranging from 11 to 20. “Severe” data represent those with symptom scores ranging from 21 to 30.

### Statistical analysis.

We compared measles, mumps, and rubella IgG titer levels from our 80 study subjects with each person’s symptom scores and severity, in both the MMR II and comparison groups. It was determined that a sample size of 29 per group would provide the trial with 80% power, using a two-sided test, alpha value set to 0.05, and a correlation value of 0.50. We exceeded the required sample size in both our MMR II group (*n* = 50) and the comparison group (*n* = 30). All statistical analyses, including the sample size calculation, were performed with the use of SAS software, version 9.2 (SAS Institute, Cary, NC). A *P* value of less than 0.05 was considered to indicate statistical significance, with adjustments made for three comparisons when measles, mumps, or rubella titer values were analyzed. All data found to be normally distributed were analyzed using a Pearson correlation coefficient, with the resulting *r* values included here. The only data that were found to not be normally distributed were the severity data, and as such, all comparisons that included severity data were done with a Spearman's rank correlation coefficient, with the resulting *r_s_* values included here. All statistical analyses were completed by Tom Vidmar (BioSTAT Consultants, Inc., Portage, MI).

### Data availability.

Deidentified individual subject data for both the MMR II group and the comparison group are available at Dryad (https://doi.org/10.5061/dryad.jsxksn077).
